# A novel system for continuous, real-time monitoring of heart motion signals

**DOI:** 10.1186/s40001-017-0252-2

**Published:** 2017-03-29

**Authors:** Guy Dori, Jorge E. Schliamser, Oscar Lichtenstein, Ilia Anshelevich, Moshe Y. Flugelman

**Affiliations:** 1 Department of Internal Medicine E, HaEmek Medical Center, Rabin Blvd, 18101 Afula, Israel; 2 Faculty of Medicine, Technion-Israel Institute of Technology, Haifa, Israel; 3 Department of Cardiovascular Medicine, Lady Davis Carmel Medical Center, Haifa, Israel; 4 Faculty of Biomedical Engineering, Technion-Israel Institute of Technology, Haifa, Israel; 5 DHS Medical Ltd., Nazareth Industrial Area, POB 1252, 17111 Nazareth Elit, Israel

**Keywords:** Cardiac motion, Cardiac twist, Cardiac rotation, Resynchronization therapy

## Abstract

**Background:**

Understanding cardiac mechanics is important for developing cardiac therapies. Current modalities for assessing cardiac mechanics sample patient’s heart at specific heart rate, contractility, preload, and afterload. The objective of this study was to test the feasibility of a novel system composed of intra-cardiac leads equipped with an inertial module chip (3D accelerometers and 3D gyroscopes) in monitoring continuous heart motion.

**Methods:**

In this descriptive study, four healthy pigs were anesthetized and instrumented with motion-sensitive intra-cardiac leads; the temporal correlation between signals from motion sensors and tissue Doppler from the chest wall were studied; changes in real-time heart accelerations (ACC) and angular velocity (ANGV) were reported as percentages of change from baseline.

**Results:**

Heart motion signals were sensed continuously from the right ventricular apex (RVa) and coronary sinus (CS). Volume expansion did not produce significant changes in the ACC and ANGV signals. Increasing heart rate increased the peak systolic ACC signal recorded from RVa and CS by 94 and 76%, respectively, and increased both peak systolic (61% RVa and 27% CS) and diastolic ANGV (200% CS vs. 31% RVa). Epinephrine administration increased peak systolic ACC signals at both sites (246% RVa; 331% CS). Peak systolic and diastolic ANGV increased in response to epinephrine (systolic: 198% RVa and 175% CS; diastolic: 723% CS and 89% RVa) (*p* = 0.125 for all changes expressed in percent). Temporal correlation between the ANGV signal and tissue Doppler signal was detected throughout all interventions.

**Conclusions:**

A novel system for continuously monitoring heart motion signals from within the heart was presented. Heart motion signals in response to physiologic manipulations were characterized.

## Background

Understanding cardiac mechanics is crucial for planning novel therapeutic devices. Unfolding the mechanics of the heart is an ongoing process, with new technologies providing important insights [1]. Pressure–volume measurement provides the corresponding data loops that describe the external work of the left ventricle (LV) and unique properties of the heart and circulation at distinct systolic and diastolic times [2]. However, such measurements do not provide information on the three-dimensional (3D) twisting–untwisting motion of the heart. Echocardiography demonstrates cardiac motion, as does cardiac MRI, which is highly informative but is resource intensive. The main limitations of echocardiography are due to the fact that the complex 3D motion of the heart is interpreted based on 2D images, and that cropped images are related to a specific clinical condition regarding heart rate (HR), blood pressure, blood volume, and medications. Newer techniques such as high-resolution echocardiography [3, 4], magnetic resonance myocardial tagging [5], speckle tracking echocardiography [6], and diffusion tensor MRI [7] were introduced to provide data regarding the unique 3D, twisting–untwisting motion of the heart. Yet, all these techniques sample heart motion during specific hemodynamic conditions. Peak endocardial acceleration (PEA) is a proprietary, accelerometer-based technology developed by Sorin (Sorin CRM SAS, Saluggia, Italy) which continuously measures myocardial contractility [8]. Yet, this technology was not reported to monitor heart motion throughout the cardiac cycle.

We hypothesized that a novel system of leads that continuously monitors heart motion from inside the heart will provide valuable physiologic information that is unattainable with current methods. In this work, we describe the feasibility of our technology in measuring continuous heart motion signals in a healthy porcine model. The complex beat-to-beat real-time cardiac motion is described as viewed from the right ventricular apex (RVa) and coronary sinus (CS), and is correlated with standard tissue Doppler (tD) signals.

## Methods

### Motion sensors and leads

**Fig. 1:**
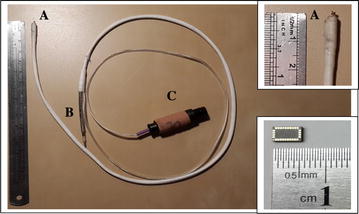
Sensor assembled on intra-cardiac lead. Bare motion sensor (*right lower corner*) and assembled sensor coated by Teflon tape on distal end of intra-cardiac lead (*right upper corner*). *A* helix at tip of lead; *B* proximal end of lead; *C* connector

The motion of cardiac walls was assessed by motion sensors (a single-chip inertial module composed of a 3D accelerometer and 3D gyroscope type LSM330D by ST microelectronics Ltd., Fig. 1, right lower corner). The sensor was glued to the distal end (Fig. 1, right upper corner, A) of a standard active fixation intra-cardiac lead (model Flextend 4088, Boston Scientific). At the tip of the distal end of the lead is an extendable/retractable spiral (A) for anchoring the lead to myocardial tissue. Sensor dimensions are 5.5 × 3.0 × 1.0 mm. The spiral can be controlled from the proximal end of the intra-cardiac lead (Fig. 1b). Conductors, coated with a thin Teflon tape that attaches them to the outer surface of the lead, connect the sensor with an evaluation board (Fig. 1c). Digital signals were transferred from the sensor to the evaluation board and to the software managing data acquisition and real-time analysis (Labview, National Instruments, Austin, Texas). Similarly, a CS lead (Deflectable decapolar catheter, Dynamic XT Bard, Lowell, MA) was prepared and used. Signals were recorded from both RVa and CS, as the former lead represents motion of the apex and the latter motion of cardiac base and atria.

### Animal preparation

The study was approved by the Institutional Animal Research committee at the Technion and all experimental procedures were performed in accordance with the guidelines for the utilization and care of animals used in research. Domestic pigs weighing 56–62 kg were anesthetized with isoflurane (2%), intubated and mechanically ventilated using room air. We studied 11 pigs. In the first pigs (*n* = 7), we established the experimental model and tested the sensing and recording systems. In four pigs, the full system was tested using the same protocol. We report the results for these four pigs.

**Fig. 2:**
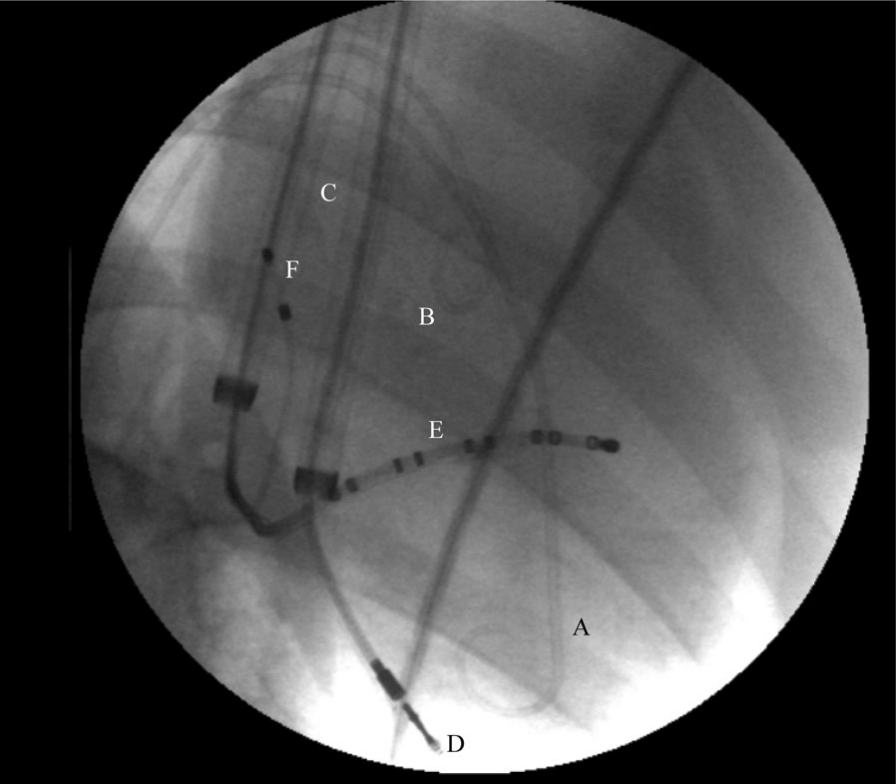
A fluoroscopic image of an instrumented animal. *A* pigtail catheter positioned in the apex of LV for measuring LV pressure; *B* pigtail catheter for measuring aortic pressure positioned in the ascending aorta; *C* two 16-Fr introducers; *D* screwing intra-cardiac lead positioned in RV apex; *E* standard intra-cardiac lead positioned in CS; motion sensors are assembled on tips of both intra-cardiac leads (not visible); *F* standard pacing lead positioned in RA. This lead was inserted from a femoral vein

After anesthesia, 16-Fr introducers were inserted into the right and left jugular veins and intra-cardiac leads tipped with 3D accelerometers and gyroscopes were positioned in the RVa and CS. Pigtail catheters were inserted through the femoral arteries into the apex of the LV and ascending aorta, for continuous measurement of pressures at these sites. A pacing lead was introduced from the right femoral vein into the right atrium (ERA 3000, pacing system, Biotronik, Berlin, Germany). Standard limb ECG was recorded throughout the study. Urine output was monitored using a urinary catheter. Figure 2 shows a fluoroscopic image (OEC 9800 PLUS Mobile C-arm, by GE Healthcare, USA) of the catheters and leads within the heart.

### Transthoracic echocardiography

All echocardiographic examinations were performed using a Vivid i machine (GE Healthcare, USA). A complete comprehensive transthoracic echocardiographic and Doppler screening examination was performed to verify normal structure and function of the heart. During the study, we recorded tD images simultaneous with heart motion and hemodynamic signals.

### Operating system

Control of sensors, data acquisition, and real-time settings (related to the process of data acquisition) were obtained using a PC that controlled a DAQ board (NI 60211, National Instruments, Texas, USA). Signals were stored and analysis was performed off-line. A mutual time-line was documented for all devices and signals.

### Experimental protocol

After the animals were prepared and instrumented, three interventions were performed to characterize motion signals of the heart. Baseline measurements were determined. Then, the effect of rapid volume loading was studied by administering 1 L of normal saline 0.9% through the left jugular introducer within 10 min. The second intervention investigated the effect of different heart rates using RA pacing at an initial rate of 10 bpm above the animals’ baseline intrinsic HR. Pulse width was 1–1.5 ms and peak amplitude 6–8 V. Pulse width and amplitude were set so as to securely induce pacing. The pacing rate was increased by 10 bpm every 2 min to a maximal HR of 130 bpm.

Third, the effects of epinephrine on both contractility and heart rate were studied by intravenously administering boluses of 0.01, 0.015, and 0.02 mg of epinephrine. Second and third boluses of epinephrine were administered after HR resumed the pre-injection rate. Ten-minute intervention-free intervals separated consecutive interventions. Reference hemodynamic, echocardiographic and cardiac motion signals were simultaneously recorded during interventions.

### Off-line analysis of motion signals

All signals were stored in a format accessible by MATLAB (The Mathworks, Massachusetts, USA). Analysis was performed using proprietary software. Heart motion signals (3 axes of ACC and 3 axes of ANGV from 2 sensors) were recorded separately. The composite signals presented throughout this work are $C_{i} = \surd \left( {X_{i}^{2} + Y_{i}^{2} + Z_{i}^{2} } \right)$ where *C* denotes the magnitude of the composite signal of *X*, *Y*, and *Z*, which are the signals recorded in each of the axes, and *i* denotes the time index. *C*_*i*_ represents ACC or ANGV. Recording *C*_*i*_ was defined as a sufficient goal, providing the magnitude of rotation in each of the twist–untwist directions. Recording and considering each of the axes of the ACC and ANGV signals would lead to dependence on the exact location and positioning of the lead. A change in the latter may result in a change in the signal. But calculating the composite lead, *C*_*i*_, overcomes this variability.

### Statistics

This study was a proof of concept and a feasibility study. To present trends of interventions, we calculated the mean (±STD) of baseline and last measurements for all animals in each of the interventions. Differences between mean baseline values (before intervention) and mean final measurement values (end of intervention) are presented in absolute and percent values. To test for significance of differences, we applied the Wilcoxon sign rank test. For all analyses performed, a *p* value <0.05 was considered statistically significant.

## Results

### Description of composite real-time heart accelerations (ACC) and angular velocities (ANGV)

**Fig. 3:**
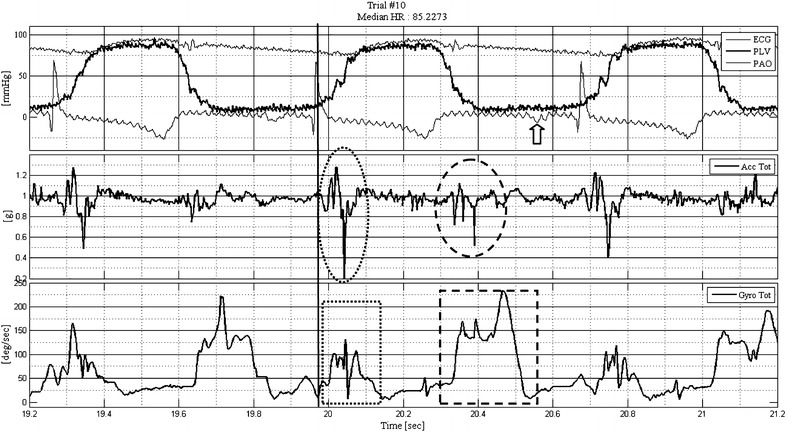
Hemodynamic, composite ACC and ANGV signals recorded from a sensor (animal 10, RVa). ECG, LV and aortic pressures are displayed in the *upper panel*. The vertical line crossing all three panels is aligned with the R wave of the second ECG complex. The *middle panel* displays the composite systolic and diastolic ACC signals (*dotted* and *dashed ellipses*, respectively). The *lower panel* displays the composite systolic and diastolic ANGV signals (*dotted* and *dashed boxes*, respectively). Filters were not used when data were recorded to avoid phase delays. *Vertical arrow* points to the second P wave. *Acc* accelerometer, *deg* degrees, *g* kg m/s^2^, *Gyro* gyroscopic signal measuring ANGV, *mmHg* millimeters of mercury, *PAO* aortic pressure, *PLV* LV pressure, *sec* second, *Tot* total or composite signal

Standard hemodynamic and heart motion signals were recorded simultaneously and showed normal physiology for the four animals: after the QRS complex, LV pressure increased to the point where the aortic valve opened and LV and aortic pressures equilibrated (Fig. 3, upper panel). Approximately at the end of the T wave, the ejection phase terminated, the aortic valve closed, and LV and aortic pressures resumed their diastolic level. The composite systolic ACC signal (Fig. 3, middle panel, dotted ellipse), which we termed systolic tension onset signal (STOS), initiated with the QRS and persisted with LV pressure rise. STOS duration was ~100 ms. Peak-to-peak amplitude (maximal to minimal value) of STOS was repeatedly greater than that of the ACC signal preceding and following it. The composite ANGV signal also started with the QRS (Fig. 3, lower panel, dotted box) and typically lasted longer than STOS (~150 ms). We note that only the composite amplitudes (not the directions) of the ACC and ANGV are presented in the graph. With the termination of the T wave and the onset of LV pressure decline, the composite ACC and ANGV signals of diastole were detected (Fig. 3, middle and lower panels, dashed ellipse and box, respectively). The duration of the diastolic ANGV signal was greater than that of the ACC signal, and this component terminated before the P wave (Fig. 3, arrow). We emphasize that LA motion is clearly sensed by CS and RVa sensors. This invaluable information of the mechanical activity of the atria is not available from ECG.

### Effect of rapid volume administration

All four animals were administered normal saline as described in the “Methods”. Peak-to-peak STOS and peak systolic and diastolic ANGV signals were not affected by rapid volume expansion (data are not shown).

### Effect of pacing (increasing heart rate)

**Fig. 4:**
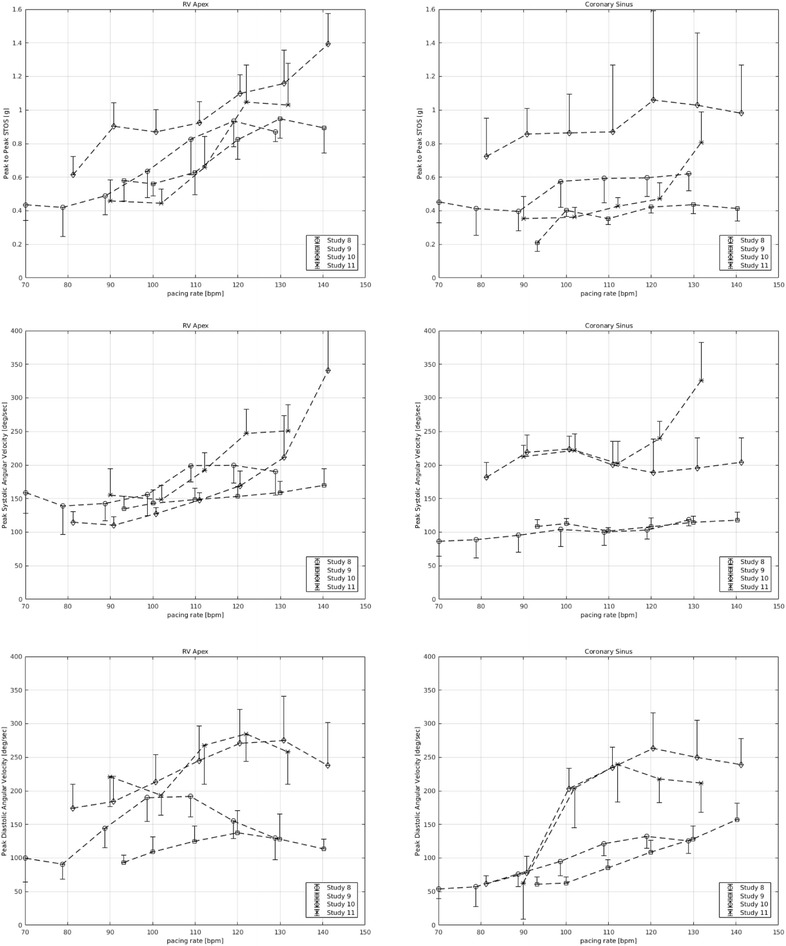
Effect of pacing. *Left and right columns* provide graphs recorded from RVa and CS, respectively. *Row 1* displays peak-to-peak STOS as a function of pacing. *Rows 2* and *3* display peak systolic and diastolic ANGV as a function of pacing, respectively. Each *point* on the graph represents a value obtained by averaging 100 consecutive values. The first icon for each animal represents the baseline value prior to pacing

Peak-to-peak STOS and peak systolic and diastolic ANGV signals increased in response to increasing heart rate (pacing). Increasing heart rate with pacing increased peak-to-peak STOS recorded from RVa and CS by 94 and 76%, respectively (*p* = 0.125) (Fig. 4, upper panels). Pacing increased both peak systolic and diastolic ANGV. The effect of increasing heart rate on peak systolic ANGV recorded from RVa was greater than that recorded from CS (61 vs. 27%; *p* = 0.125; Fig. 4, middle panels). Peak diastolic ANGV recorded from RVa increased to a maximum and then decreased for all four animals (in one animal maximal diastolic ANGV reached 110 beat/min, in two animals 120 beat/min, and in the fourth animal 130 beat/min). The diastolic ANGV signals recorded from CS showed a similar phenomenon for three animals (Nos. 8, 10, 11); while for one animal (No. 9) the signal increased with increasing heart rate (RA pacing), and did not reach a maximum. The effect of pacing on peak diastolic ANGV was greater at CS than at RVa (200 vs. 31%; Fig. 4, lower panels). The peak-to-peak STOS and the peak systolic and diastolic ANGV to higher heart rates were weaker in two animals (Nos. 8, 9) than that for the other two animals (Nos. 10, 11). Table 1, left column, summarizes the effects of pacing on STOS, and peak systolic and diastolic ANGV signals.

**Table 1:** Descriptive analysis of the trends observed during pacing and epinephrine interventions

	Pacing				Epinephrine			
	Baseline	Last measurement	Diff	Diff%	Baseline	Last measurement	Diff	Diff%
STOS-RVa	0.53 ± 0.08	1.02 ± 0.18	0.49 ± 0.15	94.5 ± 28.3	0.42 ± 0.1	1.43 ± 0.34	1.02 ± 0.27	246.04 ± 40.8
STOS-CS	0.43 ± 0.22	0.71 ± 0.25	0.28 ± 0.12	76.8 ± 46.0	0.35 ± 0.13	1.46 ± 0.35	1.11 ± 0.27	331.7 ± 90.8
Peak SAV-RVa	140.93 ± 20.4	220.4 ± 51.9	79.5 ± 62.6	60.9 ± 56.4	117.7 ± 21.2	343.1 ± 52.9	225.4 ± 58.9	198.4 ± 68.8
Peak SAV- CS	147.23 ± 59.4	190.1 ± 98.5	42.9 ± 48.2	26.9 ± 22.2	132.9 ± 69.0	317.7 ± 140.2	184.7 ± 86.0	174.8 ± 132.0
Peak DAV-RVa	147.2 ± 61.6	191.2 ± 76.3	44.1 ± 25.7	30.8 ± 12.5	138.4 ± 45.5	252.5 ± 89.7	114.1 ± 73.5	89.4 ± 59.6
Peak DAV- CS	59.8 ± 3.9	181.1 ± 56.3	121.3 ± 53.6	200.2 ± 81.4	36.7 ± 7.9	333.9 ± 293.0	297.3 ± 285.7	729.9 ± 605.3

*CS* coronary sinus, *DAV* diastolic angular velocity, *RVa* right ventricle apex, *SAV* systolic angular velocity, *STOS* systolic tension onset signal

### Effect of epinephrine

**Fig. 5:**
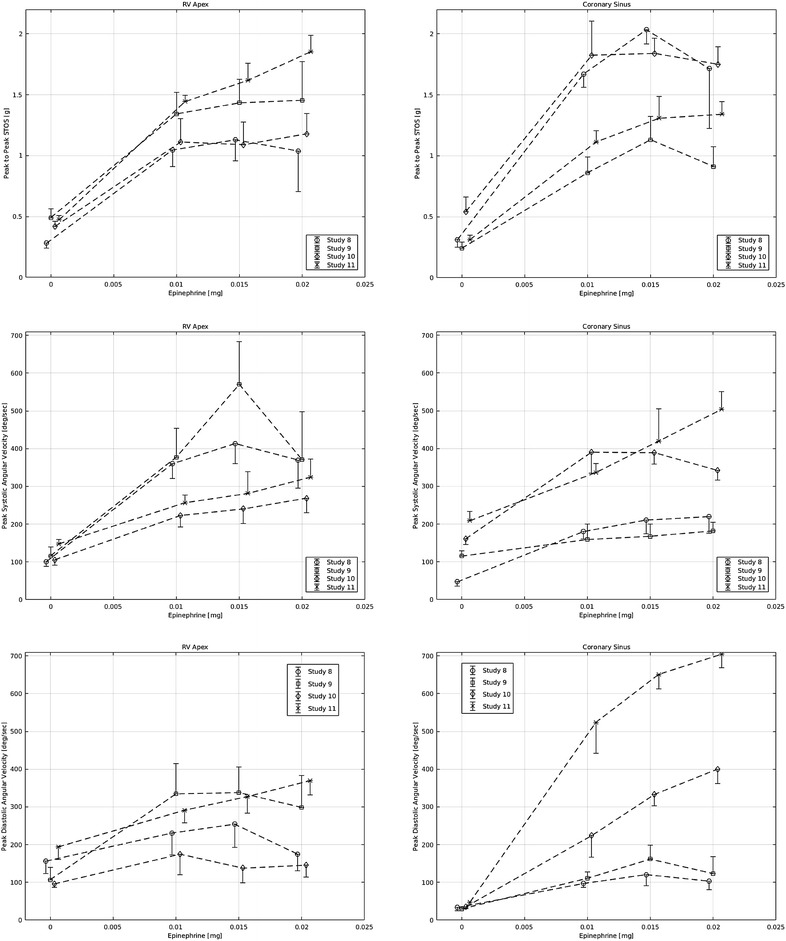
Effect of epinephrine. *Left and right columns* provide graphs recorded from RVa and CS, respectively. *Row 1* displays peak-to-peak STOS as a function of epinephrine dose. *Rows 2* and *3* display peak systolic and diastolic ANGV as a function of epinephrine dose, respectively. Every *point* in the figure represents the mean ± standard deviation of 21 points around peak epinephrine effect

All heart motion signals increased in response to epinephrine administration (Fig. 5). Peak-to-peak STOS recorded from RVa increased by 246% whereas that recorded at CS increased by 331% (*p* = 0.125). Importantly, the most prominent increases in peak-to-peak STOS recorded from RVa and CS were observed after the first dose of epinephrine (0.01 mg). Further increases in heart motion were relatively smaller. Peak-to-peak STOS recorded from CS leveled to a plateau at epinephrine boluses greater than 0.015 mg. Peak systolic ANGV recorded from RVa and CS increased in response to epinephrine administration (198 and 175%, respectively; *p* = 0.125). The effect of epinephrine administration on peak diastolic ANGV at CS was greater than that recorded from RVa (723 vs. 89%, respectively; *p* = 0.125).

Variability in the intensity of motion was observed between animals and between sites of measurement. When heart motion signals were highly intensified in response to epinephrine as recorded from RVa, the signals recorded from CS were of lower magnitude, and vice versa. Two animals (Nos. 9, 11) exhibited a greater peak-to-peak STOS response to epinephrine as recorded from RVa (compared to animals Nos. 8, 10), whereas the opposite response was discerned from the CS lead (Fig. 5, upper panels). Peak systolic and diastolic ANGV responded in a similar fashion; two animals (Nos. 10, 11) exhibited a greater peak systolic and diastolic ANGV response as recorded from CS (compared to animals Nos. 8, 9). Trends detected during epinephrine intervention are summarized in Table 1, right column.

### Temporal correlation between the ANGV signal and the standard tissue Doppler signal

**Fig. 6:**
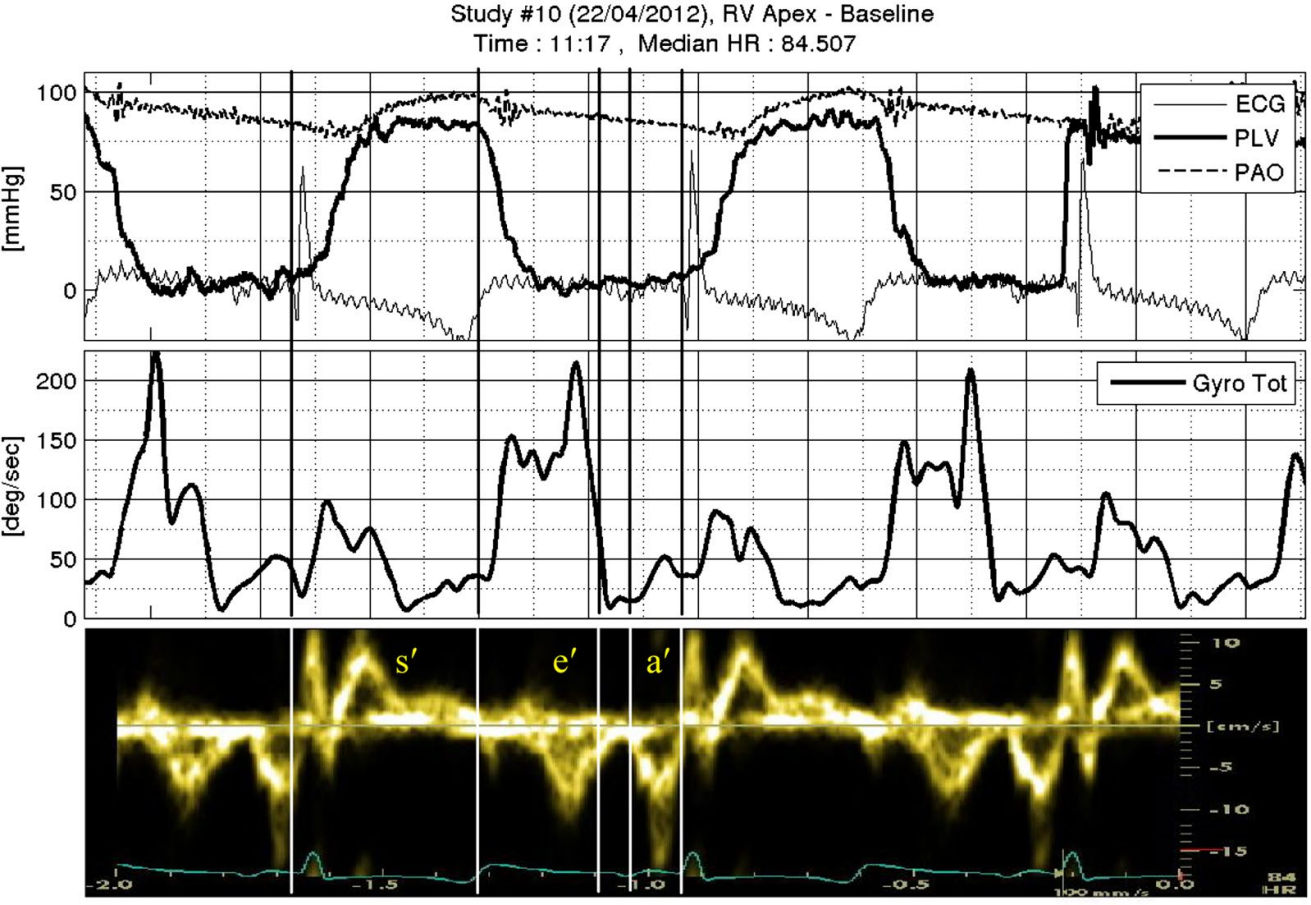
Temporal correlation between ANGV signal and tissue Doppler signal. Hemodynamic signals (ECG, LV and aortic pressures, *upper panel*), ANGV signal (*middle panel*) and tissue Doppler signal (*lower panel*) are displayed. Five vertical lines cross the three panels. Starting from left, the first line displays the onset of the ECG. The systole is confined between the first and second lines. In the *lower panel* the s′ signal is observed correlating with the systolic ANGV signal (*middle panel*). Between the second and third lines, the early rapid filling phase of diastole is observed (LV pressure decreases, *upper panel*). The correlating ANGV signal is prominent (*middle panel*) whereas the e′ signal (tD, *lower panel*) peaks in the second half of this period. Between the third and fourth lines the short diastasis can be detected, where LV pressure is almost constant, ANGV is minimal, and the Doppler signal records no tissue motion. At this period, the P wave may be observed. Between the fourth and fifth lines, the ANGV signal represents a rotation motion that correlates with the a′ signal (tD, *lower panel*)

The STOS (acceleration) and ANGV signals are novel and their full significance is yet to be investigated. However, during all studies in all animals, the temporal correlation between these motion signals and the standard tD signal recorded from the animal body surface was preserved. Specifically, ANGV signals during systole occurred at the same time as the s′ tD signal. During diastole, ANGV components occurred with e′, and a′ tD signal (Fig. 6). Though ANGV signals varied in shape between animals, the temporal correlation between the ANGV signal and tD persisted for all four animals.

## Discussion

The goal of this study was to test, on a healthy porcine model, the feasibility of a novel system for continuously measuring heart motion signals. The motion signals, ACC and ANGV, recorded from sensors, were reproducible throughout heart cycles and three physiologic manipulations. STOS reflects systolic wall tension build up in the cardiac muscle, whereas ANGV reflects systolic and diastolic velocities of twisting–untwisting motions, respectively. A signal similar to STOS, PEA, was presented by Sorin (Sorin CRM SAS, Saluggia, Italy) [8] and was used for weekly optimization of a cardiac resynchronization therapy (CRT) system. PEA provides continuous measurement of cardiac contractility, but it was not reported to monitor the twist–untwist motion of the heart. Hyler et al. [9] and Grymyr et al. [10] reported their experience with epicardial 3D accelerometer as a tool for detecting LV function in the experimental setting of post-cardiac surgery care.

ANGV signals are obtained from speckle tracking echocardiography and MRI [3–7]. However, the latter systems provide “snap-shot images” of the signals, whereas the system presented in the current study provides continuous data. Since cardiac twisting and untwisting motions must be equal in order to maintain the heart at the same position in the chest, time integrals of systolic and diastolic ANGV are also expected to be equal. These calculations were not performed as only two locations in the heart were studied, and these two sites may not necessarily record all cardiac rotational motion. ANGV may change in response to manipulations at cardiac sites that are not monitored.

Rapid *volume loading* (administration of 100 ml saline per minute for 10 min) did not produce STOS and ANGV signals that were different from baseline; therefore, conclusions may not be drawn. The volume overloading protocol that we used was insufficient to induce a significant STOS and ANGV response [11]. Hyler et al. rapidly infused 500 ml of a colloid fluid to produce an increase in tissue velocity from 14.1 to 14.8 cm/s (*p* < 0.05) [9]. Grymyr et al. used a bolus of 500 ml Ringer acetate to increase preload, yet their velocity signal (derived from the accelerometer) showed an increase in velocity at the basal area of the LV, where in contrast velocity decreased in both the basal area of the RV and LV apex [10].

During *pacing*, STOS and ANGV signals recorded from the RVa were on average greater than those recorded from the CS. This finding is hypothesized to be related to three observations: (1) the twisting trajectory taken by the RVa sensor (positioned on a lead near the cardiac apex) is larger than that by the CS sensor (positioned near the cardiac base). Previous studies showed that the rotation of the apex of the heart is greater than that of the cardiac base [1]. (2) The leverage effect on the RVa sensor is greater than that operating on the CS sensor, because the distance between the RVa sensor and an imaginary point between the aorta and the pulmonary artery (the great vessels anchoring the heart) is greater than the distance between the CS sensor and the latter anchoring point. (3) The lead and sensor positioned in the CS are limited by the anatomy of the vein, restricting their free motion. It is acknowledged that the size of the sensor attached to the lead currently limits positioning the lead more distally.

During epinephrine administration both the RVa and CS sensors responded similarly. The initial epinephrine bolus of 0.01 mg produced a prominent effect on STOS and ANGV, and subsequent higher doses had smaller incremental effects. The boluses of epinephrine used in this study forced the cardiac apex (RVa) and base (CS) to reach their maximum ability to rotate. These observations demonstrate that our system has the potential of assessing the maximal effect of an intervention on heart motion. This effect can be detected from the “Starling-like” curves such as shown in Figs. 4 and 5. This may be relevant for assessing the effects of inotropic and chronotropic medications, especially in the situation in which a drug is added to an existing regimen. Hyler et al. [9] and Grymyr et al. [10] report similar responses of the epicardial accelerometer to catecholamine administration.

This study also shows that systolic twisting and diastolic untwisting, as reflected by ANGV, may occur mainly by the apex or by the base. Our system is able to distinguish between a mainly apical or basal twist–untwist response to epinephrine.

In this work, the temporal correlation between the ANGV signals and the standard tD echocardiography was demonstrated. This correlation is predicted as both modalities sense myocardial tissue motion.

The novel system presented describes systolic and diastolic heart motion signals which reflect mechanical events of the heart cycle. As one of the goals of cardiac resynchronization therapy (CRT) is to optimize the *mechanical* synchronization between LA and LV, a mechanical event (not an electric event), i.e. ANGV signal, is proposed to be more appropriate for this purpose. Presently, the event of optimal LA emptying as the ideal timing for LV stimulation is determined using two components: (a) P wave detection and (b) a preset fixed time interval from the P wave [12]. Several factors such as normal physiologic variability and arrhythmias can lead to changes in the time from P wave detection to optimal LA emptying. Chung et al. have shown that the duration of the transmitral atrial flow velocity wave, A wave, shortens by 16 per 100% increase in HR in healthy young subjects [13]. Therefore, the method that is currently available may be inadequate as it cannot adjust the preset fixed time interval to these changes. Instead of relying on the electric system of the heart and the preset time interval, the emptying of LA can be detected directly from analysis of the LA ANGV signal (Fig. 6, middle panel, between lines 4 and 5). Using the LA ANGV signal as an indicator of LA emptying obviates the need to determine a fixed time interval. In a similar fashion, inter-ventricular delay may also be determined from systolic mechanical events, specifically LV ANGV signals recorded from RVa and CS. It must be emphasized that the above reasoning regarding CRT is merely speculative and further studies are required to validate this hypothesis. Detecting myocardial ischemia was not a goal of this work.

A significant limitation of this study was the small number of animals studied, restricting our conclusions to a level of trends. Nevertheless, we showed detectable physiological signals of mechanical movement of the heart that was reproducible and showed a tendency that reflects a novel and unique real-time record of heart movements. The ANGV and ACC signals studied were composite signals that characterize the amplitude and not the direction of motion. Signals occurred reproducibly relative to hemodynamic signals, ECG and tD, but they differed from animal to animal. For a given animal, signals remained the same throughout the study. In addition, the indices used to characterize STOS and ANGV signals, namely peak-to-peak STOS and peak ANGV, were insensitive to minor deflections of the signals. Rotation signals recorded with this experimental system should be validated using “gold standard” systems as speckle tracking echocardiography or MRI. Signals recorded from humans may differ, and must be characterized at baseline to enable assessment of the effects of physiology and pathology. Using our measuring strategy, signals were sufficiently reproducible and reliable for real-time analysis. Many issues associated with the technology remain to be studied such as calculating the exact direction of motion, differentiating cardiac motion from respiration and whole body motion, understanding regional motion abnormalities among others.

The volume overloading protocol was too mild to exert a detectable effect on the cardiac muscle. On the other hand, the effect of epinephrine administration was possibly too intense, as differences between RVa and CS leads were attenuated even with the lower epinephrine concentration.

In conclusion, the system presented provides novel real-time continuous physiologic information related to systolic and diastolic mechanical events. This information may be used to study and possibly improve the effectiveness of various therapies such as CRT; however, much research, development and validations are required.

## Appendix

### Accelerometer and gyroscope in micro-electro-mechanical systems (MEMS)

In principle, accelerometers are composed of two components: a casing upon which force operates causing it to move, and within the casing a mass component connected to the casing for example by springs. When force is applied to the casing it accelerates; the mass within the casing is displaced to the point that the spring is able to accelerate the mass at the same rate as the casing. The displacement is calibrated and measured to give the magnitude of acceleration. MEMS technology miniaturized the above principle to a microscopic level.

A mechanical gyroscope, in principle, is composed of a set of three gimbals allowing a rotor mounted on the inner most gimbal to revolve and continuously conserve the angular momentum. It is possible to measure the rotation of the outer two gimbals relative to the inner one, thereby measuring rotation. On the microscopic level (MEMS), the rotor is replaced by a vibrating element using various technologies.

## Abbreviations

3D: three-dimensional
ACC: acceleration/s in general including systolic and diastolic components
ANGV: angular velocity/ies
CRT: cardiac resynchronization therapy
CS: coronary sinus
Deg: degree
ECG: electrocardiogram
LA: left atrium
LV: left ventricle
MEMS: micro-electro-mechanical systems
PEA: peak endocardial acceleration
RV: right ventricle
Sec: second
STD: standard deviation
STOS: systolic tension onset signal
tD: tissue Doppler
